# *In vivo* imaging of palisades of Vogt in dry eye versus normal subjects using en-face spectral-domain optical coherence tomography

**DOI:** 10.1371/journal.pone.0187864

**Published:** 2017-11-27

**Authors:** Wajdene Ghouali, Rachid Tahiri Joutei Hassani, Zoubir Djerada, Hong Liang, Mohamed El Sanharawi, Antoine Labbé, Christophe Baudouin

**Affiliations:** 1 Department of Ophthalmology 3, Quinze-Vingts National Ophthalmology Hospital, Paris, France; 2 Center for Clinical Investigations INSERM 1423, Quinze-Vingts National Eye Center, Paris, France; 3 Department of Pharmacology, EA3801, SFR CAP-santé, Reims University Hospital, rue Cognacq-Jay, Reims, France; 4 INSERM, U968, Paris, France; 5 UPMC Univ Paris, UMR_S 968, Institut de la Vision, Paris, France; 6 CNRS, UMR_7210, Paris, France; 7 Department of Ophthalmology, Ambroise Paré Hospital, AP-HP, UFR Paris-île de France Ouest, University of Versailles Saint-Quentin-en-Yvelines, Versailles, France; Cedars-Sinai Medical Center, UNITED STATES

## Abstract

**Purpose:**

To evaluate a possible clinical application of spectral-domain optical coherence tomography (SD-OCT) using en-face module for the imaging of the corneoscleral limbus in normal subjects and dry eye patients.

**Patients and methods:**

Seventy-six subjects were included in this study. Seventy eyes of 35 consecutive patients with dry eye disease and 82 eyes of 41 healthy control subjects were investigated. All subjects were examined with the Avanti RTVue® anterior segment OCT. En-face OCT images of the corneoscleral limbus were acquired in four quadrants (inferior, superior, nasal and temporal) and then were analyzed semi-quantitatively according to whether or not palisades of Vogt (POV) were visible. En-face OCT images were then compared to in vivo confocal microscopy (IVCM) in eleven eyes of 7 healthy and dry eye patients.

**Results:**

En-face SD-OCT showed POV as a radially oriented network, located in superficial corneoscleral limbus, with a good correlation with IVCM features. It provided an easy and reproducible identification of POV without any special preparation or any direct contact, with a grading scale from 0 (no visualization) to 3 (high visualization). The POV were found predominantly in superior (*P*<0.001) and inferior (*P*<0.001) quadrants when compared to the nasal and temporal quadrants for all subjects examined. The visibility score decreased with age (*P*<0.001) and was lower in dry eye patients (*P*<0.01). In addition, the score decreased in accordance with the severity of dry eye disease (*P*<0.001).

**Conclusion:**

En-face SD-OCT is a non-contact imaging technique that can be used to evaluate the POV, thus providing valuable information about differences in the limbal anatomy of dry eye patients as compared to healthy patients.

## Introduction

The limbal palisades of Vogt (POV) have been proposed as the site of the stem cell niche [1]. Limbal epithelial stem cells play a major role in the corneal epithelium homeostasis [2] and are thus essential in dry eye physiopathology [3].

In clinical practice, the limbal region is difficult to assess. Slit-lamp examination does not precisely analyse the POV and the changes that may occur in limbal stem cell deficiency (LSCD). In vivo confocal microscopy (IVCM) can visualize POV at the cellular level and has been described as a method to evaluate the human limbus and the diagnosis of LSCD [4,5]. However, despite good resolution and good vizualisation of cells morphology and cellular integrity of POV, this method is operator-dependent, difficult and limited due to the restriction of the scan area.

Optical coherence tomography (OCT) is a noninvasive imaging modality that uses interference of light to perform a high-resolution cross-sectional study of biological samples [6]. Another approach appeared with the use of en-face technology that compiles many transversal scans to provide frontal images [7].This imaging modality enables the assessment of various ocular surface diseases, most particularly conjunctiva/cornea tissue analysis [8,9]. This technology can also provide a good visualization of POV in the limbal region with no preparation or direct contact with the limbus [10,11].

The purpose of this study was to determine a reproducible and reliable evaluation of the corneoscleral limbus with en-face SD-OCT and to compare certain features in normal eyes and eyes with dry eye disease.

## Patients and methods

### Subjects

A total of 76 subjects (152 eyes) were enrolled. This study was conducted at the Center of Clinical Investigations (CIC 503) at the Quinze-Vingts National Ophthalmology Hospital, Paris, France, with the approval of the Institutional Review Board of Saint-Antoine University Hospital (CPP Ile-de-France 5, national agreement 10793) and in accordance with the ethical principles stated in the Declaration of Helsinki.

All subjects signed informed consent at the inclusion.

Eighty-two eyes of 41 healthy volunteers were enrolled as per the following inclusion criteria: no history of ocular disease and no signs or symptoms of ocular surface disease.

Seventy eyes of 35 dry eye patients were enrolled. Dry eye was defined as complaints of ocular irritation associated with objective clinical signs: Schirmer 1 testing < 10 mm at 5 min, tear film instability and interpalpebral ocular fluorescein staining (at least 2 on the Oxford Scheme) [12].

Exclusion criteria for both groups were the following: age <18 years, contact lens wear, previous eye surgery and current or use within the last 6 months of eye drops (other than nonpreserved tear substitutes for the dry eye group)

### Ophthalmologic examination

Medical history and demographic information were collected for each patient. Then they underwent a detailed ophthalmic examination with anterior segment and fundus examination. Dry eye patients were asked to complete the Ocular Surface Disease Index (OSDI) questionnaire and underwent a specific examination of the ocular surface in both eyes as follows: tear film break-up time (TBUT), conjunctival and corneal fluorescein staining using the Oxford scheme (graded from 0 to 5) and a Schirmer test without anesthesia. Finally, considering signs and symptoms of dry eye disease, a severity grading score (1–4) was determined for each patient according to the 2007 Dry Eye Workshop. Moderate dry eye was defined by a score of 1 or 2, and severe dry eye was defined by a score of 3 or 4.

### Anterior-segment optical coherence tomography imaging

A spectral-domain OCT (Avanti RTVue-XR, Optovue Inc., Fremont, CA, USA) with a corneal adaptor module was used in this study. This device works at an 840-nm wavelength and provides 70,000 axial scans per second, thus providing 5-μm axial and 15-μm transverse resolution. All images were acquired by a single trained operator.

For each patient, high-resolution scans with cross-sectional and en-face acquisitions were made across the central cornea and the limbal area in four quadrants (inferior, superior, nasal and temporal). After acquisition of a high-density SD-OCT volume scan with a 3×3-mm^2^ area, the 3D data were processed to generate C-scans or en-face OCT in the coronal plane, while B-scans (conventional OCT) were derived from transverse sections. C-scans were automatically determined by two boundaries parallel to the surface. The distance between these two boundaries corresponded to the C-scan thickness. The default value of this thickness was 31μm. The C-scan was manually swiped to explore the acquired volume from the surface to the depth. Images were acquired ~70 μm below the corneal/conjunctiva surface.

### Image analysis

**Fig 1:**
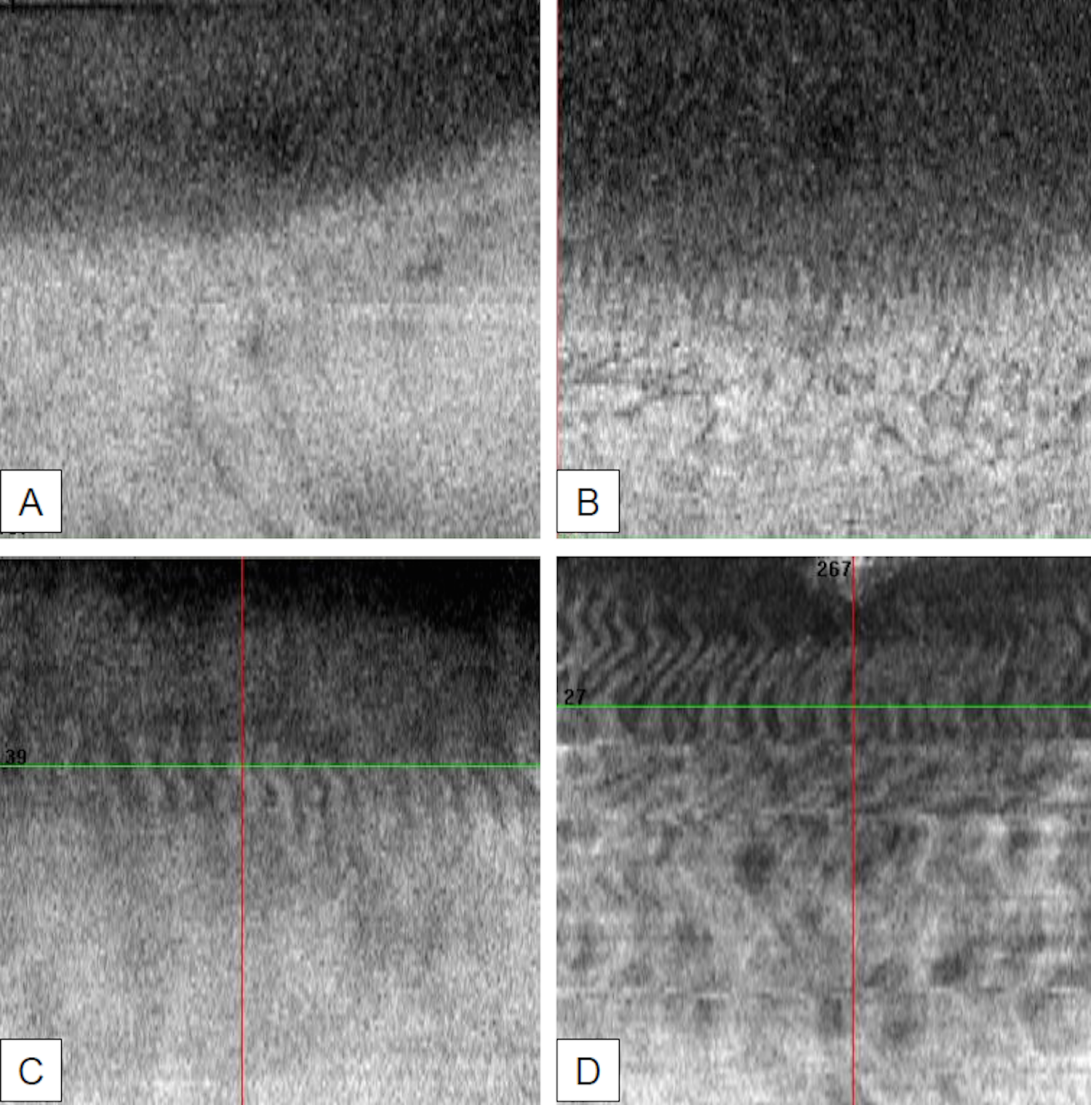
En-face OCT limbal features with corresponding grade. Palisades of Vogt are clearly identified as a network of radially oriented, channel-like and high reflective features located in the superficial corneoscleral limbus. A shows grade 0 (no visualization of palisades of Vogt) B shows grade 1 (low visualization of palisades of Vogt) C shows grade 2 (intermediate visualization of palisades of Vogt) D shows grade 3 (high visualization of palisades of Vogt).

A semi-quantitative OCT grading scale of the POV, based on their degree of visibility, was proposed by two corneal specialists (Fig 1). The corneal specialists were not given access to the patients' data.

To evaluate the reproducibility of this classification, two observers were masked to the subject's ophthalmologic examination and status, and then independently asked to classify the OCT scans according to the grading system. To evaluate intra- and interobserver reproducibility, the images were analyzed a second time 1 month later.

### In vivo confocal microscopy imaging

Eleven eyes of 7 healthy and dry eye patients were then imaged with in vivo confocal microscopy. IVCM imaging was performed with the Heidelberg Retina Tomograph II/Rostock Cornea Module (Heidelberg Engineering GmbH, Heidelberg, Germany) confocal microscope. A drop of topical anesthesia with 0,4% oxybuprocaine and a drop of gel tear substitute were instilled before examination. During acquisitions, x-y position of images and the section depth were controlled manually. The images obtained consisted of two-dimensional high resolution optical sections covering an area of 400*400 μm, with 2 μm transversal resolution and 4 μm longitudinal optical resolution. IVCM examination was performed in the central cornea and in the limbal area four quadrants. All images were acquired by a single trained operator.

**Fig 2:**
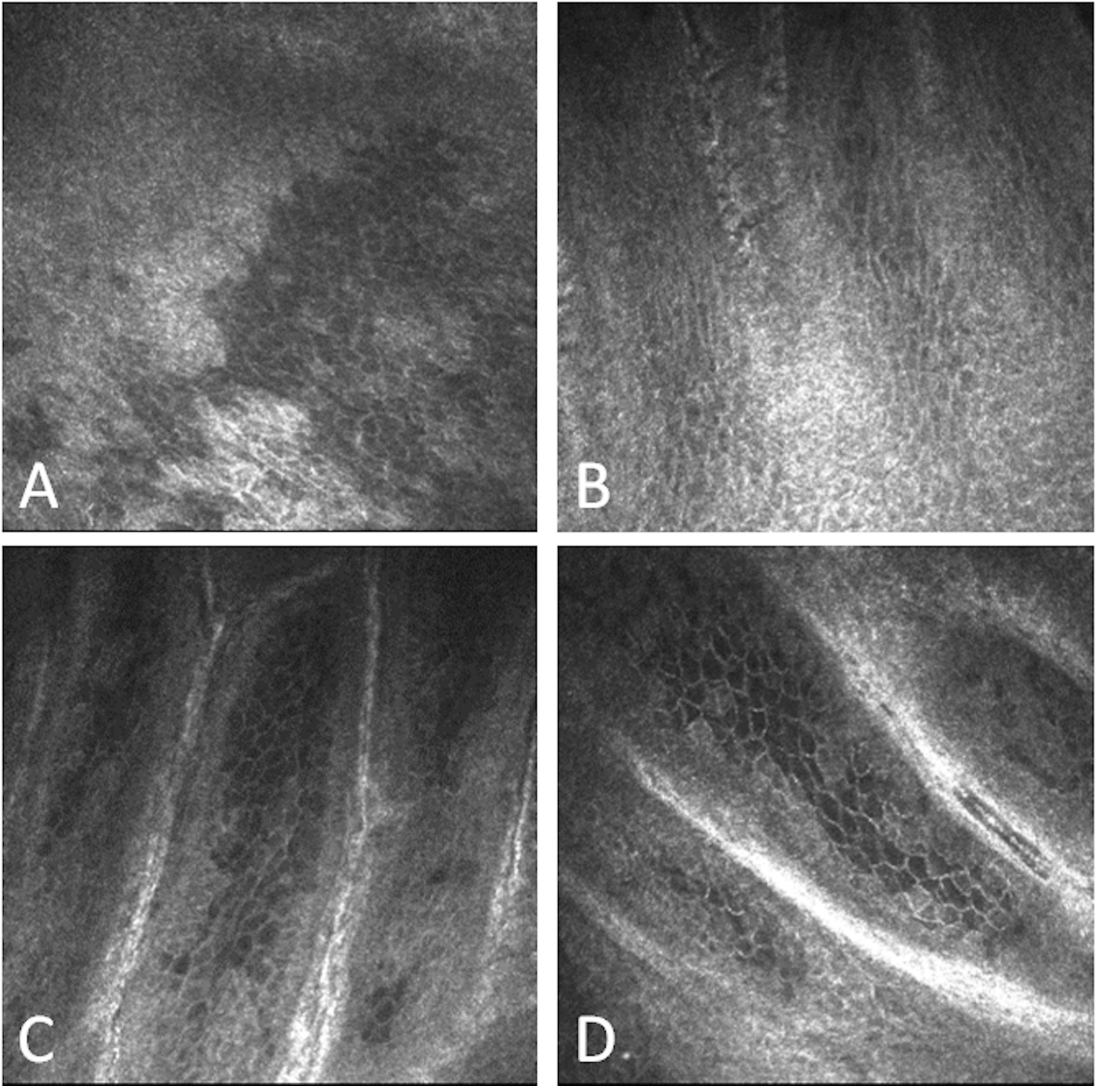
IVCM limbal features with corresponding grade. Palisades of Vogt are clearly identified as a network of radially oriented, channel-like and high reflective features located in the superficial corneoscleral limbus. A shows grade 0 (no visualization of palisades of Vogt) B shows grade 1 (low visualization of palisades of Vogt) C shows grade 2 (intermediate visualization of palisades of Vogt) D shows grade 3 (high visualization of palisades of Vogt).

As for en-face OCT, a semi-quantitative IVCM grading scale of POV, based on their degree of visibility, was used (Fig 2).

En-face OCT and IVCM scores were then compared.

### Statistical analysis

The data were analyzed using SPSS software (SPSS, Inc., Chicago, IL, USA) version 20.0 [13].

With regard to the OCT limbal staging, agreement between observers was assessed using a Kruskal-Wallis test. Intraobserver reproducibility was checked through a Wilcoxon matched pairs test. The measurement of observer (inter- and intraobserver) agreement for OCT grade was also assessed by kappa statistics. Differences between groups were evaluated using the Kruskal-Wallis test. If the *P*-values were significant, Dunn's Multiple Comparison Test was applied to assess the significance between different groups. For qualitative variables, univariate analyses were performed using the chi-square test.

Factors influencing the visualization of POV (age, gender, location, presence of dry eye disease, presence of severe dry eye disease) were assessed through a multivariate analysis using the multinomial regression modeling. *P*-values of 0,05 or less were considered statistically significant.

The measurement of agreement between en-face OCT and IVCM scores was assessed by kappa statistics.

## Results

### Patient demographics

A total of 76 patients (31 males and 45 females) were enrolled in this study. The mean patient age was 43.57 ± 18.29 [range, 19–79] years. Forty-one healthy volunteers and 35 dry eye patients were included in the study. No significant differences in baseline characteristics (age and gender) between the two groups were apparent (*P* = 0.27 for age, *P* = 0.64 for gender). In the dry eye group (*n* = 35), there were 16 moderate dry eye subjects and 19 severe dry eye subjects: the OCT severity grading score was grade 1 in eight subjects, grade 2 in eight subjects, grade 3 in nine subjects and grade 4 in ten subjects.

### Anatomic description

Image sets acquired with SD-OCT and reconstructed with C-mode imaging provided visualization of POV. These structures appeared as a network of radially oriented, channel-like and high reflective features located in the superficial corneoscleral limbus (Fig 1). These features were comparable to those observed in IVCM (Fig 2).

### En-face OCT limbal grading scale

The established en-face OCT limbal grading scale was based on the degree of POV visibility from 0 to 3. In grade 0, there was no visualization of any radially oriented and channel-like structures. In grade 1, there was a blurred visualization of radially oriented structures that was unclear. In grade 2, there was an intermediate visualization with a few clear radially oriented structures. Finally, in grade 3, there was a high visualization of radially oriented structures with clear stripes (Fig 1).

En-face SD-OCT and IVCM images scores were correlated with a substantial agreement (kappa value equal to 0,69 with P<0,001)

### Reproducibility of the en-face OCT grading scale of the POV

When comparing the OCT grading scale of the POV for normal and dry eye patients in all quadrants, we observed no difference between the two observers' measurements (at least *P*>0.2). Similarly, no intraobserver difference was found (at least *P*> 0.2). The kappa value for intraobserver grading was equal to 0.8 (*P*<0.0001) demonstrating perfect agreement. The kappa value for interobserver grading was equal to 0.72 (*P*<0.0001), demonstrating substantial agreement.

### Factors associated with POV visualization on C-scans

**Fig 3:**
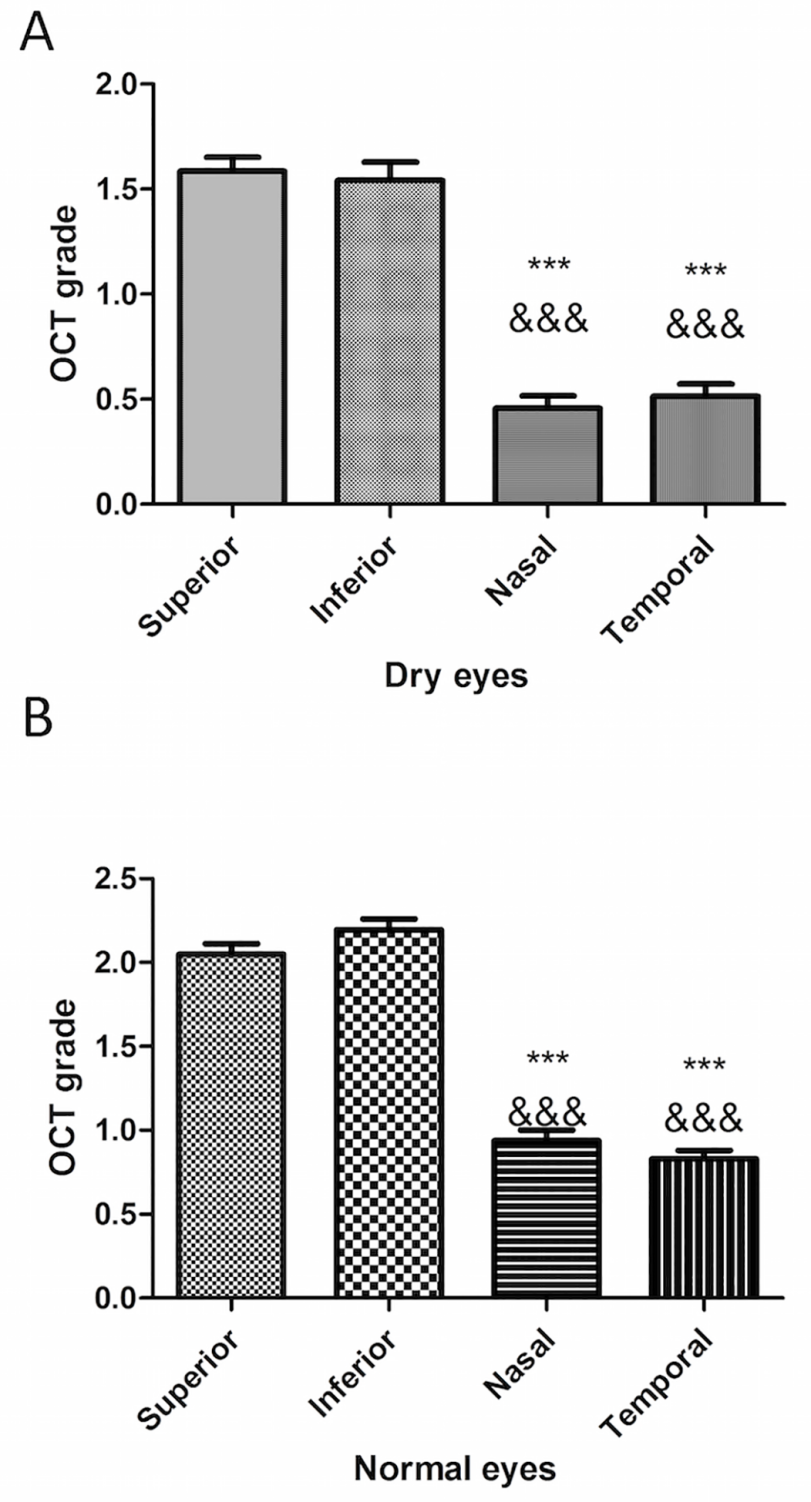
**Impact of location of the visualization on OCT grade in dry (A) and normal (B) eyes.** Data are means ± standard error of the mean. Differences between groups were evaluated using the Kruskal-Wallis test. If the *P*-values were significant, Dunn's Multiple Comparison Test was applied to assess significance between different groups. *** *P*< 0.001, compared to superior visualization group; &&&*P*< 0.001, compared to inferior visualization group.

**Fig 4:**
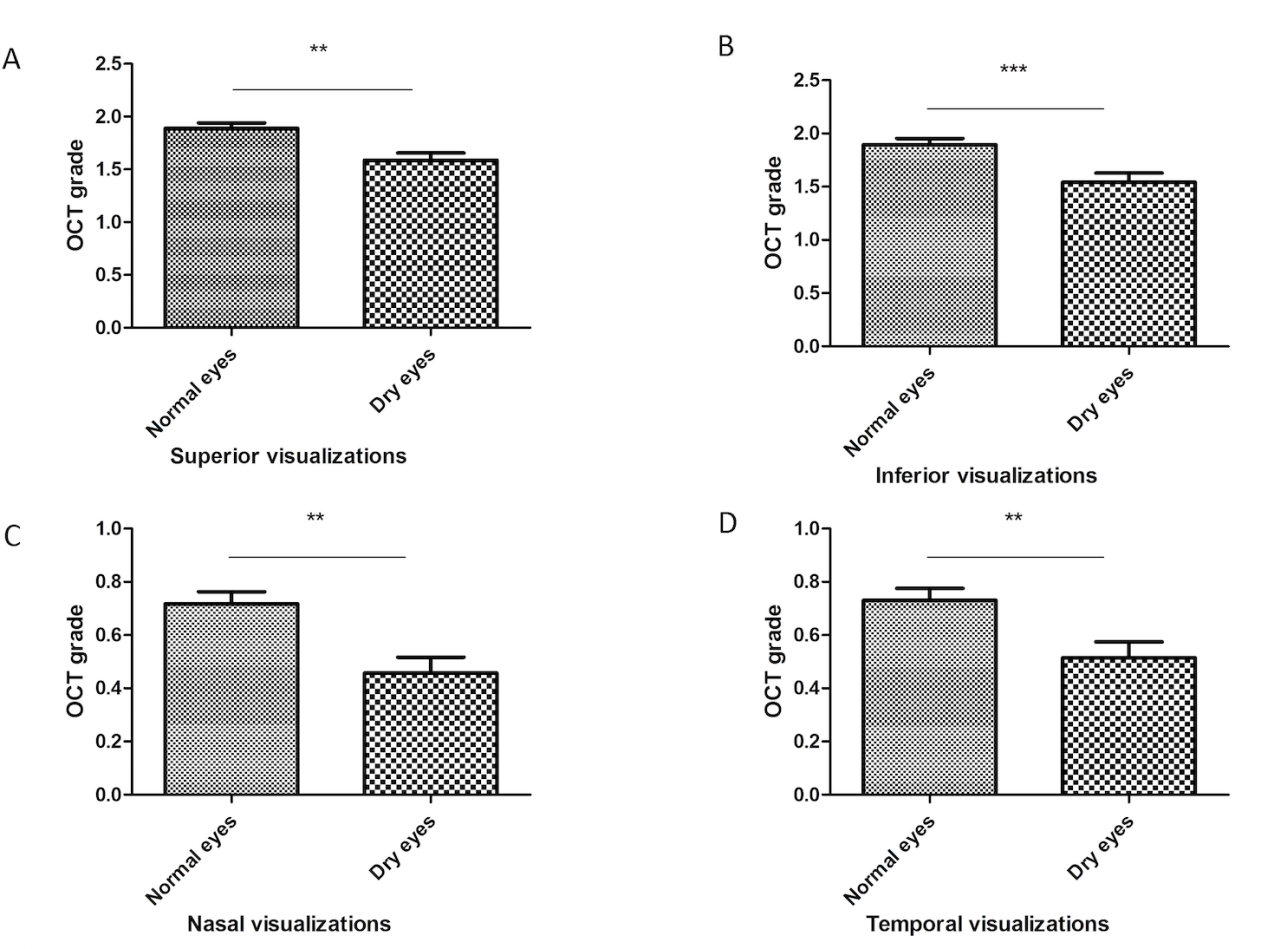
**Difference in OCT grade between dry and normal eyes considering the location of visualization (superior A, inferior B, nasal C and temporal D).** Data are means ± standard error of the mean. Differences between groups were evaluated using the Mann-Whitney test. ** *P*< 0.01, compared to normal eye group; ***P< 0.001, compared to normal eye group.

Multivariate multinomial regression analysis was carried out to determine the factors positively or negatively influencing the POV visibility score (Table 1). In dry eye patients (Fig 3A), the location influenced the visualization of POV, which were significantly better visualized in the inferior quadrant and superior quadrants (*P*<0.001). In normal subjects (Fig 3B), the same tendency was also found with significant differences. Similarly, POV were better viewed in young subjects (*P*<0.001, Table 1). In all quadrants, fewer POV were detected in dry eye patients (Fig 4A–4D) (*P*<0.01 for all quadrants when compared to normal subjects).

**Table 1:** Association between OCT score and different factors using multivariate multinomial regression analysis.

OCT score				95% Confidence interval	
	Factor	*P*-value	Odds ratio	Lower limit	Upper limit
0–1	Severity of dry eye disease	<0.0001	0.293	0.177	0.488
	Age	<0.0001	0.93	0.911	0.95
	Dry eye disease	0.028	0.25	0.072	0.864
	Inferior location	<0.0001	62	14.3	>100
	Superior location	<0.0001	>100	22.67	>100
0–2	Severity of dry eye disease	<0.0001	0.058	0.027	0.123
	Age	<0.0001	0.868	0.842	0.896
	Dry eye disease	0.002	0.066	0.012	0.364
	Inferior location	<0.0001	>100	>100	>100
	Superior location	<0.0001	>100	>100	>100
0–3	Severity of dry eye disease	<0.0001	0.008	0.001	0.049
	Age	<0.0001	0.787	0.751	0.826
	Dry eye disease	0.004	0.018	0.001	0.275
	Inferior location	<0.0001	>100	>100	>100
	Superior location	<0.0001	>100	>100	>100

Multivariate multinomial analysis shows factors positively or negatively influencing the limbal feature visibility score.

Table 2 compares the visibility of POV in normal eyes, moderate dry eye disease and severe dry eye disease. The severe dry eye patients (score, 3–4) presented the lowest scores when compared to the moderate dry eye patients (score, 1–2) in all quadrants (*P*<0.001 for four quadrants). However, the moderate dry eye patients presented no difference in POV visibility when compared to the normal subjects.

**Table 2:** Difference in OCT grade between dry and normal eyes considering location of visualization and severity of dryness.

Location of visualization	Normal eyes	Moderate dry eye disease (score, 1 and 2)	Severe dry eye disease (score, 3 and 4)
Superior visualization	2.05 ± 0.06	1.81 ± 0.09	1.29 ± 0.10***^;^^&&^
Inferior visualization	2.19 ± 0.06	2.00 ± 0.11	1.21 ± 0.10***^;^^&&&^
Nasal visualization	0.94 ± 0.06	0.69 ± 0.08	0.18 ± 0.06***^;^^&&&^
Temporal visualization	0.83 ± 0.05	0.63 ± 0.09	0.29 ± 0.07***^;^^&^

Data are means ± standard error of the mean. Differences between groups were evaluated using the Kruskal-Wallis test. If the *P*-values were significant, Dunn's Multiple Comparison Test was applied to assess significance between different groups.

***P < 0.001, compared to normal eye group

& *P* < 0.05, compared to dry eye group (severity: 1, 2)

&& *P*< 0.01, compared to dry eye group (severity: 1, 2)

&&& *P* < 0.001, compared to dry eye group (severity: 1, 2).

## Discussion

The stem cell niche is difficult to assess. Previous studies have thoroughly described the limbus using IVCM [4,5]. POV appear as hyper-reflective linear acellular structures alternating with columns of epithelial cells. IVCM analyzes the limbus at the cellular level. Although it cannot specifically identify limbal stem cells within the POV, IVCM well identifies the POV which is accepted to constitute the stem cell niche. However, IVCM is difficult to practice because it is operator-dependent and is restricted to an observation area limited to 400×400 μm.

For OCT analysis, only two publications have described limbal features using en-face OCT images [10,11]. In our study, we used a commercially available device and proposed a newly established SD-OCT grading scale of of the POV. Despite its macroscopic description, this classification was reproducible, confirming the reliability of OCT limbal images by different ophthalmologists. En-face OCT features were comparable to those observed in IVCM. Furthermore, IVCM and en-face OCT limbal scores were correlated, suggesting the possibility of using en-face OCT for the assessment of the limbal area.

IVCM imaging of the cornea was first described in 1985 by Lemp et al [4]. This imaging technique allows en-face optical sections through the full thickness of the living cornea, providing in addition quantitative analysis of the layers, nerves and cells. Improvements of SD-OCT technology lead to an increasing use of this imaging modality, which perfoms high-resolution cross-sectionnal study of the cornea in a transveral plane. Thus, IVCM and SD-OCT are often jointly used in the assessment of anterior segment diseases, providing complementary information. IVCM allows to study cellular integrity of POV, morphology of cells and smaller structures such as focal stromal projections, which is not possible with OCT. So at present, OCT can give an idea of POV presence and density, but not ‘quality’ of the structures.

Table 3 summarizes the differences between OCT B-scans, en-face OCT and IVCM. Few studies reported the use of en-face OCT for the assessment of anterior segment of the eye [8–11]. We first described a correlation between limbal IVCM and en-face OCT features, thus offering a new way to assess the POV in normal and dry eye patients.

**Table 3:** Comparison of B-scan OCT, en-face OCT and IVCM.

	B-scan OCT	En-face OCT	IVCM
Plan of analysis	Transversal	Transversal	Frontal
Area of analysis	Adjustable from 1*1 mm to 8*8 mm	Adjustable from 1 to 8 mm	400*400 μm
Examination mode	Non-contact	Non-contact	Contact
Image acquisition	Easy, with minimal experience	Easy, with minimal experience	Quite difficult and operator-dependant
Axial resolution	5 μm	5 μm	2 μm
Transversal resolution	15 μm	15 μm	4 μm

After analyzing the limbal features in the four quadrants, we provided reliable information on a possible heterogeneity of the limbal stem cell niche location. POV were statistically better viewed in superior and inferior areas. Our findings were in accordance with a previous IVCM study of the limbus: Shortt et al. showed a predominance of limbal structures in the superior and inferior quadrants, whereas the nasal and temporal structures could not be identified [14]. Interestingly, *in vitro* studies have confirmed that the limbal stem cell niche was more present in the superior and inferior regions [14]. Our results were consistent with these findings and suggest that the stem cell niche is predominant in regions covered by the lids. Lids would indeed play a protective role against light and external aggressions that would damage the cornea and the limbal regions. For instance, pterygium, a condition where the cornea is invaded by a fibrotic tissue from the conjunctiva, usually occurs in the nasal limbus [15]. The paucity of limbal epithelial stem cells in the nasal side could play a role in the predominance of pterygia on this location.

Considering the influence of age, we also showed that limbal features were better visualized in young subjects. In slit-lamp biomicroscopy studies of the limbus, POV were not observed in 10–20% of the population, especially in nonpigmented and in older subjects [16]. A previous IVCM study showed age-related changes in human limbus, with a decreasing tendency of the positive rate of POV with age [16].

Zheng and Xu observed age-related changes of blood vessels, suggesting an influence of age on serum-derived modulation, which is known to play an important role in niche regulation of limbal epithelial stem cells [16]. Moreover, they showed a reduction of melanocyte density with aging. The stem cell niche contains melanin-pigmented cells that play the role of protecting stem cells from UV irradiation [17]. The decrease of melanocytes could thus affect the density of limbal epithelial stem cells with aging.

This study also assessed the limbal features in patients with dry eye disease by showing a decreased pattern of POV in dry eye patients. The integrity of the stem cell niche is essential for maintenance and regeneration of the corneal epithelium [18,19]. The dry eye condition is known to induce chronic inflammation and epithelial turnover [20]. Levels of inflammatory cytokines in tears and infiltration of CD4+ T cells at the limbus [21] may thus affect the function or the integrity of the limbal stem cells. Previous IVCM studies have shown a corneal conjunctivalization in patients with severe dry eye disease [22], suggesting that functional or morphological damage of the stem cell niche could result in various degrees of LSCD diseases. Interestingly, we observed differences in the limbal OCT score depending on the degree of eye dryness. In moderate dry eye patients, there was no difference in visibility when compared to normal subjects. We hypothesize that in moderate eye dryness, factors leading to inflammation [23] disrupt the niche regulation of corneal epithelial stem cells at the limbus but do not affect the integrity of POV. On the other hand, our results showed very low POV visibility in severe dry eye patients when compared with moderate dry eye disease or normal subjects. This finding could be explained by a real degradation of POV in severe and advanced dry eye disease, as previously described in studies concerning the limbus deficiency in dry eye [22,24]. The low visibility of POV in severe dry eye disease could also be explained by a low quality of imaging acquisition due to the opacification of superficial layers by metaplastic and/or inflammatory epithelia induced by reduced aqueous tear production [25]. An *ex vivo* model of squamous metaplasia induced by dryness showed that abnormal epithelial differentiation emerged only from the stem-cell containing limbal, but not corneal, epithelium without intrinsic alteration of stem cells in the limbus [26]. These data suggest that dry eye disease could be responsible of a functional LSCD.

Our findings seem to be consistent with previous b-scan SD-OCT studies that evaluated and graded the alterations of ocular surface epithelia *in vivo*. Francoz et al. analyzed the epithelial thickness along the limbus and found a thicker epithelium in superior and inferior limbal quadrants, and thinner epithelium in patients with dry eye disease [27]. In the same way, Liang et al. showed that these changes were correlated to the severity of dry eye symptoms and tear film alterations [28].

In clinical practice, an easy method of imaging POV, thus possibly reflecting the limbal stem cell niche, would be very useful for diverse ocular surface diseases. As previously reported [8], the main advantages of en-face SD-OCT, when compared to IVCM or other clinical tools, were reproducibility, rapidity of image acquisition and the noninvasiveness of this technology. En-face OCT was easily performed with minimum experience required. In comparison to IVCM, OCT images of this study were acquired at lower resolution to afford a larger field. Thus, the different quadrants of the corneoscleral limbus areas could easily be analysed.

The characterization of the structure and distribution of the stem cell niche could help target limbal biopsies in cases of autografts in LSCD, thus obtaining higher yields of limbal epithelial stem cells in culture [14]. En-face OCT of the limbus could also help assess the efficacy of medical and surgical treatments in the management of LSCD by tracking temporal changes in the aspects of POV. Finally, the assessment of the anatomical status of the limbal region would allow the follow-up of many ocular conditions that may affect the limbal region such as dry eye disease, contact lens wear or ocular surface surgery.

However, several limitations encountered in this study need to be highlighted. Firstly, OCT limbal images were not correlated with limbal slit-lamp features. We believe that the visualization of POV with slit-lamp biomicroscopy is highly inconstant. Secondly, en-face OCT findings should be combined with immunostaining of targeted tissues to confirm the hypothesis that en-face OCT limbal features are the reflection of the limbus stem cell niche structure and function. Thirdly, the depth of analysis chosen in our study (70 μm below the corneal/conjunctiva surface) might not capture the entire POV structure. However, this depth was used in previous en-face OCT and IVCM studies [4,10] and prevents variation of reflectivity of POV according to depth, thus allowing a reproducibility of measures. Finally, further studies on larger populations of dry eye patients are required to assess the correlation between the POV visibility grading scale proposed herein and the severity or duration of the other ocular surface disease. IVCM currently remains the gold standard to obtain information such as vascular changes or cell size, inflammatory infiltrates and morphological characteristics. A preliminary optical coherence tomography angiography study successfully assessed anterior segment vasculature [29]. Further studies and improvements in OCT technology will probably lead to a cellular approach and therefore a better understanding of limbal changes in normal and diseased conditions.

## Supporting information

S1 Table Analysis Dr T 1. (XLS)

S2 Table Analysis Dr T 2. (XLS)

S3 Table Analysis Dr H 1. (XLS)

S4 Table Analysis Dr H 2. (XLS)
